# The CD8 T Cell Response to Respiratory Virus Infections

**DOI:** 10.3389/fimmu.2018.00678

**Published:** 2018-04-09

**Authors:** Megan E. Schmidt, Steven M. Varga

**Affiliations:** ^1^Interdisciplinary Graduate Program in Immunology, University of Iowa, Iowa City, IA, United States; ^2^Department of Microbiology and Immunology, University of Iowa, Iowa City, IA, United States; ^3^Department of Pathology, University of Iowa, Iowa City, IA, United States

**Keywords:** CD8 T cell, memory T cell, respiratory virus, respiratory syncytial virus, influenza A virus, human metapneumovirus, rhinovirus, coronavirus

## Abstract

Humans are highly susceptible to infection with respiratory viruses including respiratory syncytial virus (RSV), influenza virus, human metapneumovirus, rhinovirus, coronavirus, and parainfluenza virus. While some viruses simply cause symptoms of the common cold, many respiratory viruses induce severe bronchiolitis, pneumonia, and even death following infection. Despite the immense clinical burden, the majority of the most common pulmonary viruses lack long-lasting efficacious vaccines. Nearly all current vaccination strategies are designed to elicit broadly neutralizing antibodies, which prevent severe disease following a subsequent infection. However, the mucosal antibody response to many respiratory viruses is not long-lasting and declines with age. CD8 T cells are critical for mediating clearance following many acute viral infections in the lung. In addition, memory CD8 T cells are capable of providing protection against secondary infections. Therefore, the combined induction of virus-specific CD8 T cells and antibodies may provide optimal protective immunity. Herein, we review the current literature on CD8 T cell responses induced by respiratory virus infections. Additionally, we explore how this knowledge could be utilized in the development of future vaccines against respiratory viruses, with a special emphasis on RSV vaccination.

## Introduction

Given its continuous exposure to the outside environment, the respiratory mucosa is highly susceptible to viral infection. The human respiratory tract can be infected with a variety of pulmonary viruses, including respiratory syncytial virus (RSV), influenza virus, human metapneumovirus (HMPV), rhinovirus (RV), coronavirus (CoV), and parainfluenza virus (PIV) (1). The severity of disease associated with respiratory viral infection varies widely depending on the virus strain as well as the age and immune status of the infected individual. Symptoms can range from mild sinusitis or cold-like symptoms to more severe symptoms including bronchitis, pneumonia, and even death. RSV is the leading cause of severe lower respiratory tract infection in children under 5 years of age (2). RSV is commonly associated with severe lower respiratory tract symptoms including bronchiolitis, pneumonia, and bronchitis and is a significant cause of hospitalization and mortality in children, the elderly, and immunocompromised individuals (2–6). Similarly, PIV commonly infects children and is a major cause of croup, pneumonia, and bronchiolitis (7, 8). Seasonal influenza infections, most often of the influenza A virus (IAV) subtype, are responsible for 3–5 million cases of severe infection annually (9). Seasonal IAV infections also result in approximately 290,000–650,000 deaths per year, most commonly in either young children or elderly populations (9–11). However, infection with emerging pandemic IAV strains, such as the 2009 H1N1 pandemic strain, primarily induces severe disease and mortality in otherwise healthy adults younger than 65 years of age (12). In contrast, respiratory infection with HMPV, RV, and CoV are most commonly associated with symptoms of the common cold (13–15). Two notable exceptions are severe acute respiratory syndrome (SARS) CoV and Middle East respiratory syndrome (MERS) CoV, which cause acute respiratory distress and mortality in infected individuals (16–18).

Despite their profound impact on human health, most common respiratory viruses lack an approved vaccine. The strategy employed most often in vaccine development is the induction of robust neutralizing antibody responses. However, the hallmark of many respiratory viral infections, including RSV, HMPV, and RV, is the ability for reinfections to occur frequently throughout life (19–21). This suggests that the antibody response to these respiratory viruses may wane over time. Indeed, despite a correlation between pre-existing nasal IgA and protection from reinfection, the development of long-lasting RSV- and RV-specific mucosal IgA responses was poor in infected adults (22, 23). Although IAV-specific neutralizing antibodies are elicited efficiently through either infection or vaccination, IAV vaccine formulations must be redeveloped annually to account for the rapid mutations of HA and NA genes in seasonal strains (24). Therefore, vaccinations that solely promote the induction of neutralizing antibodies may not be optimal in providing protection against many respiratory virus infections. The induction of cellular immune responses has thus far received little attention in respiratory virus vaccine development. CD8 T cells play a critical role in mediating viral clearance following many respiratory virus infections including RSV, IAV, and HMPV (25–27). In addition, recent murine studies utilizing CD8 T cell epitope-specific immunization strategies observed significantly reduced lung viral titers following IAV, RSV, or SARS challenges (28–30). Therefore, the induction of virus-specific CD8 T cell responses has the potential to improve upon the efficacy of current vaccination strategies. Here, we review the current literature on CD8 T cell responses following respiratory virus infections and discuss how this knowledge may best be utilized in the development of future vaccines.

## CD8 T Cell Responses to Acute Respiratory Virus Infections

Following an acute respiratory infection, dendritic cells (DCs) that have taken up viral antigen stimulate the activation of naive CD8 T cells in the lung draining lymph node to induce robust virus-specific CD8 T cell responses [reviewed in Ref. (31–33)]. Respiratory virus infection in mouse models results in an increase in the frequency and number of total and antigen-specific CD8 T cells in the lungs and airways. RSV-specific CD8 T cell responses typically reach peak numbers in the lung at approximately day 8 following an acute infection (34–37). The kinetics of virus-specific CD8 T cells are slightly more delayed following other respiratory virus infections with peaks occurring at approximately day 10 for IAV, days 10–14 for HMPV, and days 12–14 for pneumonia virus of mice (PVM), a model respiratory virus (38–42). The peak of the antigen-specific CD8 T cell response generally corresponds to lung viral clearance following RSV, IAV, HMPV, and PVM infections (36, 40–43). Human CD8 T cell kinetics following respiratory virus infections are less well known given the lack of identified CD8 T cell epitopes and the difficulty in obtaining respiratory tract samples from children following initial virus exposure. The frequency of total activated CD8 T cells in tracheal aspirates peaked at approximately 10 days after the onset of symptoms in children with RSV, IAV, RV, or CoV infections (44). RSV-specific CD8 T cells were detected in the tracheal aspirates of children; however, the evaluated epitopes were present at very low frequencies, comprising up to only 2% of the total CD8 T cell response (44). Following peak expansion, CD8 T cell contraction occurs and a memory population of virus-specific CD8 T cells remains within the lung. The majority of virus-specific CD8 T cells are located within the lung parenchyma, rather than the pulmonary vasculature, following localized respiratory infections in mice (37). Similarly, human RSV- and IAV-specific CD8 T cells were enriched within the lung compared to the blood (45). RSV-specific CD8 T cells in tracheal aspirates of children remain elevated during convalescence following a severe RSV infection, in contrast to murine studies (46). These studies suggest that CD8 T cell responses in the airways may be more prolonged following viral clearance in humans compared to mice.

Following respiratory virus infection, CD8 T cells become activated and develop the ability to produce inflammatory cytokines. Virus-specific CD8 T cells in the lung and airways of mice upregulate expression of markers associated with activation including CD11a, CD25, NKG2a, and CD44 as well as downregulate expression of the lymphoid homing receptor CD62L (34, 35, 47, 48). Activated CD8 T cells also acquire effector functions following viral infection. Virus-specific CD8 T cells rapidly produce cytokines including IFN-γ and TNF as well as degranulate, as measured by CD107a expression, following *ex vivo* peptide stimulation (35, 38, 41, 48). Human virus-specific CD8 T cells also acquire an activated phenotype and effector functions following a respiratory virus infection. CD8 T cells from the tracheal aspirates of children following RSV, RV, or CoV infections expressed elevated levels of the activation markers CD38 and HLA-DR and the proliferation marker Ki-67 (44). Expression of effector molecules such as granzyme B and perforin were also increased. Similarly, CD8 T cells from bronchiolar lavage (BAL) fluid samples exhibited increased expression of Ki-67, granzyme B, CD38, and HLA-DR following either experimental RSV infection of adults or severe, natural RSV infection of infants (46, 49). Additionally, human virus-specific CD8 T cells produce cytokines following respiratory virus infection, as peripheral blood CD8 T cells secreted IFN-γ, TNF, and IL-2 following stimulation with peptides derived from RSV, IAV, HMPV, or RV (49–53).

Following contraction, a subset of virus-specific CD8 T cells remain in the host to form a long-lasting memory population that provides protection against subsequent infection. CD8 T cell contraction to form long-term memory populations in the lung is regulated in part by inflammatory chemokine signaling (54). Mice deficient in either CXCR3 or CXCR3 and CCR5 exhibit a significant increase in the number of memory CD8 T cells following IAV infection, suggesting that chemokine signaling through CXCR3 and CCR5 plays a critical role in T cell memory generation (54). Following respiratory viral infections in mice and humans, virus-specific CD8 T cells can be detected up to several months post-infection (47, 49, 55, 56). However, respiratory virus-specific memory CD8 T cell populations decline in magnitude with age in the peripheral blood (57). Interestingly, adult RSV-specific CD8 T cell responses are significantly reduced compared to IAV-specific CD8 T cell responses in the peripheral blood, suggesting that memory CD8 T cell responses to IAV in humans may be more stable than RSV (57). Memory CD8 T cells rapidly expand in the lung following a secondary respiratory virus infection in both mice and humans (35, 38, 39, 44, 49). The observed expansion is primarily due to the migration of circulating CD8 T cells into the lung and airways, rather than proliferation of resident cells (58). The expansion of virus-specific CD8 T cells in the lung and airways following infection corresponds with an increase in CXCR3- and CCR5-binding chemokines, supporting a role for chemokine-mediated migration of CD8 T cells following secondary infection (59). Indeed, CCR5 expression on memory CD8 T cells is required for their early recruitment into the airways after secondary infection, but not to the lung parenchyma (59). Following secondary expansion, memory CD8 T cells rapidly produce effector cytokines such as IFN-γ and TNF (30, 38, 60). Additionally, virus-specific memory CD8 T cells express high levels of CD11a and produce cytolytic molecules, such as granzyme B, after infection (61, 62). These effector functions of respiratory virus-specific memory CD8 T cells are critical for mediating viral clearance and protecting against infection, as discussed below.

Based on the expression of activation marker CD45RA and lymphoid homing receptor CCR7, human memory CD8 T cells have been broadly separated into four major subsets: (1) naive (CD45RA^+^CCR7^+^), (2) central memory (T_CM_; CD45RA^-^CCR7^+^), (3) effector memory (T_EM_; CD45RA^−^CCR7^−^), and (4) late effector memory (T_EMRA_; CD45RA^+^CCR7^−^) (63). Due to their expression of CCR7, T_CM_ home primarily to secondary lymphoid organs, while T_EM_ migrate to peripheral tissues and rapidly exert effector functions. T_EMRA_ are a subset of T_EM_ cells that have re-expressed CD45RA. They exhibit reduced proliferative and functional capacity, and thus are considered to be terminally differentiated cells. Human virus-specific memory CD8 T cell populations are typically composed of a combination of T_EM_ and T_EMRA_ within the peripheral blood (44, 46, 50, 52, 55). Alternatively, RSV-specific memory CD8 T cells located in the airways in both adults and infants are primarily of T_EM_ phenotype and also express high levels of CD27, CD28, and CCR5 and low levels of CD62L (46, 49). Together, these studies indicate that T_EM_ CD8 T cells are dominant following respiratory virus infection in humans. Given the frequent exposure to viruses in the respiratory tract, T_EM_ cells may be critical for the rapid employment of CD8 T cell effector mechanisms following reinfection.

Recently, an additional population of memory CD8 T cells that persist within peripheral tissues has been identified, termed tissue-resident memory CD8 T cells (T_RM_) (64). T_RM_ have been observed within several peripheral organs including the intestine, skin, female reproductive tract, and lung. T_RM_ generated following a respiratory virus infection represent a non-circulating population of memory CD8 T cells that are maintained within the lung parenchyma (65). Virus-specific T_RM_ are located along the wall of large airways and within pulmonary tissue surrounding bronchioles and alveoli (66, 67). Respiratory virus infection also induces T_RM_ within the airway lumen (68, 69). Airway T_RM_ downregulate CD11a expression and can be distinguished from recently trafficked CD8 T cells that express high levels of CD11a (70, 71). The localization of lung and airway T_RM_ following respiratory virus infection is distinctly different from that of T_CM_, which traffic through the pulmonary vasculature and accumulate in the lung-draining lymph node (72, 73). Virus-specific T_EM_ are also differentially located from T_RM_ residing primarily in the pulmonary vasculature or within the lung tissue near blood vessels, spacially distinct from regions that contain T_RM_ (67, 73). Following either RSV or IAV infection in mice, lung and airway T_RM_ are induced and can be identified by their expression of the canonical resident memory markers CD69 and CD103, which promote their migration to and retention within the lung tissue (37, 65, 74–76). IAV- and RSV-specific T_RM_ are also generated in the lungs of mice that have been locally vaccinated via an intranasal route, but not mice that have been immunized systemically (30, 77–79). Importantly, IAV-specific T_RM_ expressing CD69 were also detected in human lung tissue sections but were absent from the spleen (65). Similarly, RSV-specific T_RM_ expressing both CD69 and CD103 were identified in the human BAL but were not present in the peripheral blood (49). Following secondary viral infection, T_RM_ expand prior to the recruitment of circulating memory CD8 T cell populations from the peripheral blood and rapidly produce IFN-γ (60, 66). Thus, T_RM_ provide a crucial first-line of defense for protecting the host from re-infection with a respiratory virus. However, in contrast to other memory CD8 T cell subsets that remain stable for long periods of time, IAV-specific T_RM_ exhibit limited longevity and enhanced apoptosis with time following infection (66, 80). The loss of IAV-specific T_RM_ corresponds to an increase in viral titers and weight loss following a heterosubtypic IAV infection (66, 80). Interestingly, infant mice generate fewer lung T_RM_ following IAV infection or vaccination and exhibit reduced heterosubtypic protection compared to mice initially infected as adults (79). Given the role of lung T_RM_ in providing protection against respiratory virus infections, identifying strategies to promote the generation of long-lived T_RM_ will be critical for future vaccines, particularly for infant populations.

## Mechanisms of CD8 T Cell-Mediated Viral Clearance

It has been well established that CD8 T cells are critical for viral clearance following an acute respiratory virus infection in mice. Adoptive transfer of CD8 T cell clones resulted in significantly reduced viral titers in the lung following RSV, IAV, or HMPV infections (25, 27, 81–84). Similarly, the transfer of either RSV- or IAV-immune splenic CD8 T cells accelerated viral clearance in the lung following infection (85–89). Accordingly, RSV infection of mice depleted of CD8 T cells resulted in significantly increased lung viral titers at day 7 post-infection, although the virus was ultimately cleared by day 11 (26). In contrast, depletion of CD8 T cells alone did not alter clearance of HMPV (40). Instead, depletion of both CD4 and CD8 T cells together elevated lung virus titers at day 7 following infection with HMPV. Importantly, CD8 T cells have been shown to be sufficient to mediate viral clearance in the lung following acute respiratory infections (87, 88). Athymic nude mice, which lack T cells, fail to clear either RSV or IAV resulting in persistent infections (87, 90). However, the transfer of either RSV- or IAV-immune splenic CD8 T cells into athymic mice resulted in significantly reduced lung viral titers by day 15 and 21, respectively (87, 88). Together, these studies indicate that CD8 T cells play a critical role in mediating viral clearance following acute respiratory infections in mice.

Although studies are limited, a role for CD8 T cells in the elimination of respiratory viruses has also been established in humans. Early studies demonstrated that immunocompromised children with T cell defects experienced prolonged viral shedding following RSV, IAV, or PIV infections compared to immunologically normal children (3, 91, 92). Following bone marrow transplantation of an RSV-infected child with severe combined immunodeficiency, a marked reduction in nasal viral load was observed that correlated with an elevation of CD8 T cell counts (92). Recently, it has been demonstrated that the number of pre-existing virus-specific CD8 T cells in the airway of adults experimentally infected with RSV correlated with reduced overall viral load in the nasal cavity and bronchial brushings (49). In addition to pre-existing CD8 T cell numbers, CD8 T cell effector functions also correlate with reduced viral load. CD8 T cell target cell lysis activity measured by chromium-release assay correlated with a lack of viral shedding in the nasal washes of adults experimentally infected with H1N1 IAV (93). Additionally, individuals with the lowest frequencies of IFN-γ^+^ CD8 T cells exhibited the highest viral titers following natural H7N9 IAV infection (94). These studies support the role of CD8 T cells in respiratory virus clearance in humans, consistent with the numerous murine studies.

CD8 T cells mediate viral clearance by utilizing a variety of effector mechanisms to induce the apoptosis of virus-infected cells (95). CD8 T cells can use direct cell–cell contact to eliminate target cells through the interactions of surface molecules such as Fas (CD95) and FasL (CD95L). Additionally, TRAIL expressed on CD8 T cells can interact with its receptors DR4 and/or DR5 to induce the destruction of infected cells. CD8 T cells can also secrete perforin and granzymes to cause membrane pore formation and induce apoptosis. Lastly, CD8 T cells produce inflammatory cytokines, such as IFN-γ and TNF, which may either directly or indirectly promote the cell death of virus-infected cells. While the exact mechanism utilized is unclear, many of these effector functions have been associated with CD8 T cell-mediated clearance of respiratory viruses. Fas/FasL interactions and the perforin pathway have been established as the primary mechanisms by which CD8 T cells eliminate infected cells following an IAV infection (96, 97). Studies utilizing TRAIL-deficient mice and antibody-mediated TRAIL blockade have also demonstrated a role for TRAIL in CD8 T cell-mediated clearance of IAV (98, 99). Similarly, Fas/FasL and perforin pathways have also been associated with virus elimination following RSV infection. Perforin-deficient and FasL-deficient *gld* mice exhibit significantly delayed viral clearance (100, 101). However, both perforin-deficient and *gld* mice achieve complete viral clearance by day 10 post-infection, suggesting that CD8 T cells compensate for those deficiencies through alternative mechanisms. One such mechanism is likely TNF production, as neutralization of TNF in perforin-deficient and *gld* mice significantly increased viral titers compared to IgG-treated controls (101). This is in contrast to studies following PVM and IAV infections, where viral clearance occurs independently of TNF (102, 103). IFN-γ does not appear to play a prominent role in CD8 T cell-mediated viral clearance, as both IFN-γ-deficient mice and mice that received IFN-γ-deficient CD8 T cells exhibit equivalent viral titers to wild-type mice following RSV, PVM, or IAV infections (101, 103–105). Together, these studies demonstrate that CD8 T cells use multiple complementary mechanisms to eliminate virally infected cells following a respiratory virus infection.

## CD8 T Cells Protect Against a Secondary Infection

Given the ability of CD8 T cells to mediate viral clearance following an acute viral infection, it is no surprise that memory CD8 T cells also play a critical role in protecting against secondary respiratory virus infections. The adoptive transfer of airway IAV-specific memory CD8 T cells resulted in significantly reduced lung titers following IAV challenge compared to PBS transfer controls (60). Similarly, transfer of airway RSV-specific memory CD8 T cells reduced lung viral load and weight loss following subsequent RSV infection (76). These studies indicate that transferred memory CD8 T cells are capable of providing protection against secondary respiratory virus challenge.

Memory CD8 T cell-mediated protection against secondary infection has been shown more convincingly in mouse models through the use of vaccination strategies to generate virus-specific memory CD8 T cells. Recombinant baculovirus or murine cytomegalovirus (MCMV) vectors expressing the RSV M2 protein induced M2-specific CD8 T cells that mediated the reduction of lung viral titers following RSV challenge (78, 106). Whole protein vaccination with HMPV virus-like particles containing F and M proteins elicited HMPV-specific CD8 T cells that reduced viral titers in μMT mice, which lack antibodies (107). CD8 T cell epitope vaccines against either RSV or HMPV have also demonstrated CD8 T cell-mediated protection following challenge by reducing lung viral load and histopathology compared to unimmunized controls (108, 109). A similar strategy utilizing DC-peptide vaccination to generate pre-existing PVM-specific memory CD8 T cells also resulted in enhanced viral control following PVM infection (42). Recently, several studies have utilized DC-prime, recombinant *Listeria monocytogenes*- (DC-LM) or vaccinia virus-boost (DC-VV) vaccination protocols to generate a high frequency of pre-existing antigen-specific memory CD8 T cells in the absence of virus-specific CD4 T cell memory and antibodies (28–30). Prime-boosted mice exhibited significantly reduced lung viral titers following RSV, IAV, or SARS infections compared to controls lacking virus-specific memory CD8 T cells. Additionally, memory CD8 T cells were able to reduce weight loss and mortality following lethal challenges with either IAV or SARS. Overall, these studies provide clear evidence that memory CD8 T cells provide protection against secondary respiratory virus infection by reducing viral titers.

The most well studied example of memory CD8 T cell-mediated protection from a secondary respiratory virus infection is heterosubtypic immunity to IAV subtypes. IAV-specific neutralizing antibody responses recognize the surface glycoproteins hemagglutinin (HA) and neuraminidase (NA), which vary between subtypes as a result of genetic reassortment, known as antigenic drift. However, the internal proteins of the virus are often conserved between IAV subtypes. Therefore, memory CD8 T cells that recognize epitopes within conserved viral proteins may be capable of providing cross-protection between IAV viruses of differing subtypes. Evidence of heterosubtypic immunity was first demonstrated by the protection of H1N1 IAV-immune mice from a lethal H2N2 IAV challenge without the induction of a neutralizing antibody response (110). Since then, a memory CD8 T cell-mediated role in accelerating clearance of a heterosubtypic IAV strain has been well-established in mouse, chicken and non-human primate models (66, 111–114). Recently, it has been demonstrated that T_RM_ are essential in providing cross-protection against secondary IAV infection with a heterosubtypic strain (60, 66, 80). Mice with CD103^+^ T_RM_ in the lung exhibit more efficient viral clearance and reduced weight loss following heterosubtypic challenge than mice lacking a T_RM_ response (66). Importantly, the protection was provided solely by lung-resident memory CD8 T cells, as blocking the ability of recently proliferated T_CM_ cells from trafficking to the lung did not impact protection (66). Consistent with the limited lifespan of IAV-specific T_RM_, heterosubtypic protection by memory CD8 T cells wanes over time, with a decline observed as early as 60 days following the initial infection (80, 111). Interestingly, systemic immunization with cognate antigen is capable of boosting the T_RM_ pool by expanding the circulating T_EM_ population that seeds the lungs (80). Therefore, it is possible that T_RM_-mediated heterosubtypic protection could be re-established by vaccination after a waning of the protective T_RM_ population in the lung.

While protection in mouse models is well established, whether memory CD8 T cells play a critical role in protection following secondary respiratory infection in humans is currently unclear. Similar to studies in murine models, evidence for heterosubtypic immunity mediated by memory CD8 T cells has also been demonstrated in humans. Individuals lacking H1N1-specific neutralizing antibody titers exhibited an inverse correlation between memory CD8 T cell activity and viral shedding following their first exposure with H1N1 IAV (93). More recently, it was demonstrated that the frequencies of pre-existing cross-reactive memory CD8 T cells correlated with reduced symptoms, including fewer patients with fever, sore throat, and cough, following infection with the 2009 pandemic H1N1 IAV strain (50). Similarly, a correlation between pre-existing H3N2-specific memory CD8 T cells and reduced risk of viral shedding following 2009 pandemic H1N1 IAV infection was observed (115). Thus, memory CD8 T cell-mediated heterosubtypic protection is also likely to be critical in humans. Following experimental RSV infection in humans, the frequency of pre-existing RSV-specific memory CD8 T cells in the airways correlates with a reduction in both cumulative and lower respiratory tract symptom scores, suggesting a possible role for memory CD8 T cells in protection against RSV in humans (49). However, evidence has also been provided suggesting that memory CD8 T cells may not contribute to protection following respiratory virus infections in humans. Natural reinfection of infants with RSV did not result in a boosting of the CD8 T cell response (116). Similarly, the frequency of RSV-specific memory CD8 T cells in the peripheral blood of healthy adults is significantly reduced compared to IAV-specific memory CD8 T cells (57). Therefore, the extent to which memory CD8 T cells play a role in providing protection against RSV infection in humans remains unclear.

## CD8 T Cell-Mediated Immunopathology Following Respiratory Virus Infection

Despite their beneficial role in mediating viral clearance and protecting against secondary infection, CD8 T cells have also been associated with the induction of immunopathology following respiratory virus infection. Although mice depleted of CD8 T cells exhibited elevated lung viral titers, weight loss and symptom illness scores were significantly reduced in CD8 T cell depleted mice following acute RSV infection (26). Similarly, the adoptive transfer of CD8 T cell lines exacerbated weight loss following an acute RSV infection, despite accelerating viral clearance (82–84). Similar reduction in disease severity was also demonstrated following either HMPV or PVM infection of CD8 T cell depleted mice or mice genetically deficient in CD8 T cells, respectively (40, 103). In addition to the induction of immunopathology following acute respiratory virus infections, we recently demonstrated that memory CD8 T cells also mediate severe immunopathology following secondary RSV infection (30). Large frequencies of systemic, pre-existing RSV-specific memory CD8 T cells generated through DC-LM immunization induced significant weight loss, pulmonary dysfunction, and mortality following RSV challenge, despite a significant reduction in lung viral titers. This result was in contrast to studies using similar immunization strategies to either IAV or SARS, in which memory CD8 T cells mediated protection against lethal viral challenge in the absence of immunopathology (28, 29). Interestingly, the immunopathology induced by RSV-specific memory CD8 T cells occurred only in the context of an RSV infection, as mice challenged with a recombinant IAV strain expressing an RSV-derived CD8 T cell epitope exhibited significantly reduced morbidity and were protected from mortality (30). This result is consistent with several studies that demonstrate CD8 T cells enhance viral clearance while preventing mortality following IAV infection (25, 81, 85, 87, 117). Together, these studies demonstrate a clear role for CD8 T cells in the development of immunopathology following primary and secondary infections with some respiratory virus infections, particularly RSV.

Antiviral mechanisms utilized by CD8 T cells to mediate viral clearance following respiratory virus infection also contribute to the development of immunopathology. Removal of the Fas/FasL pathway in *gld* mice resulted in significant amelioration of weight loss and symptom illness scores following RSV infection (101). Similarly, RSV-infected perforin-deficient mice exhibited prolonged weight loss and symptom illness scores compared to wild-type mice (100). TNF contributes substantially to immunopathology, as antibody-mediated depletion of TNF prior to either RSV or IAV infection significantly reduced weight loss (101, 102, 118). Additionally, mice that are IFN-γ-deficient, depleted of IFN-γ, or received an adoptive transfer of IFN-γ-deficient CD8 T cells prior to RSV infection lost significantly less weight than controls (89). CD8 T cell production of TNF and IFN-γ following PVM infection also induced pulmonary immunopathology by initiating a cytokine storm (119). In addition to causing disease in acute respiratory infections, IFN-γ produced by memory CD8 T cells mediated the severe and fatal immunopathology following RSV infection of DC-LM prime-boosted mice (30).

The role of CD8 T cells in the development of pathology following respiratory infections in humans remains unclear. The best evidence supporting a pathogenic role for CD8 T cells in humans infected with respiratory viruses comes from a study evaluating an RSV-infected severe-combined immunodeficiency patient after bone marrow transplantation (92). The patient exhibited increased CD8 T cell counts following bone marrow transplant, which corresponded to a sharp reduction in RSV nasal titers. However, the appearance of CD8 T cells also correlated with a marked increase in respiratory rate indicative of reduced pulmonary function. Also supporting a pathogenic role of CD8 T cells is the finding that children requiring mechanical ventilation due to severe RSV infection expressed significantly increased levels of activated granzymes and more CD8 T cells producing granzyme B compared to healthy controls (120). In contrast, a study of infants following either fatal IAV or RSV infections revealed a near absence of CD8 T cells from affected lung regions by immunohistochemical staining (121, 122). Similarly, infants with severe RSV infection exhibited an underexpression of genes related to CD8 T cells in the peripheral blood (123). In support of a protective, rather than pathogenic, role of CD8 T cells, correlations have been identified between increased CD8 T cell cytolytic activity and cytokine production with reduced symptom score, faster recovery, and fewer fatalities following H1N1 or H7N9 IAV infections (93, 94). Therefore, whether CD8 T cells play a primary role in mediating pathology versus protection following human respiratory virus infection remains controversial and is an important topic of future investigation.

## Regulation of CD8 T Cell Effector Functions

Given the potential of CD8 T cell effector functions to cause immunopathology following respiratory virus infection, the immune system has evolved critical regulatory mechanisms to prevent prolonged CD8 T cell effector activity following viral clearance. CD8 T cell effector functions, including production of IFN-γ and TNF, are suppressed in the lung following the resolution of IAV and RSV infections (124–127). One of the primary mechanisms utilized to limit the CD8 T cell response is through suppression by regulatory CD4 T cells (Tregs). Tregs accumulate in the lungs following either RSV or IAV infection peaking at approximately day 6 post-infection, prior to the peak of the CD8 T cell response (36, 128–130). Antibody-mediated depletion of CD25^+^ Tregs prior to RSV infection resulted in exacerbated weight loss, pulmonary dysfunction, and lung inflammation (128). This enhanced illness corresponded to an increased frequency of RSV-specific CD8 T cells and elevated levels of IFN-γ and TNF protein in the lung (36, 128). Consistent with the Treg depletion studies, increasing RSV-specific Tregs prior to RSV infection using RSV peptide-immunization resulted in an amelioration of weight loss and a reduction in CD8 T cell numbers in the blood and spleen, but not the lung (131). Tregs also can suppress CD8 T cell effector functions following a secondary infection with IAV (130). Antibody-mediated CD25^+^ Treg depletion prior to heterosubtypic IAV challenge resulted in enhanced inflammation and pulmonary dysfunction corresponding to an increase in CD8 T cell numbers and IFN-γ production. One mechanism through which Tregs may suppress CD8 T cell responses is through the production of the anti-inflammatory cytokine IL-10. FoxP3^+^ Tregs secrete IL-10 following primary infection with RSV or IAV (130, 132–134). Infection of either IL-10-deficient mice or mice treated with IL-10 receptor blocking antibody resulted in increased numbers of either IFN-γ^+^ or IFN-γ^+^TNF^+^ CD8 T cells, suggesting that IL-10 suppresses CD8 T cell effector functions following respiratory virus infection (132–134). Interestingly, IL-10 production by FoxP3^−^ CD4 T cells and CD8 T cells following either RSV or IAV infection has also been reported, indicating that effector T cell responses may self-regulate their effector functions (132–135). Together, these studies demonstrate that Tregs and IL-10 production play a critical role in regulating CD8 T cells following primary and secondary respiratory virus infections to prevent immunopathology.

Interactions between inhibitory receptors on CD8 T cells with their ligands represents another important mechanism mediating the inhibition of CD8 T cell effector functions following infection. Regulation of CD8 T cells through the PD-1:PD-L1 pathway is a common inhibitory pathway utilized following respiratory virus infection. Expression of PD-1 on pulmonary CD8 T cells is upregulated following RSV, IAV, or HMPV infection in mice (41, 136, 137). Blockade of PD-L1 in primary RSV, IAV, or HMPV and secondary HMPV infections results in enhanced CD8 T cell effector functions, including IFN-γ, TNF, and granzyme B production (41, 136–138). CD8 T cell effector functions are also enhanced following either HMPV or IAV infections in PD-1-deficient mice (41). Importantly, the PD-1:PD-L1 pathway has also been associated with human CD8 T cell responses. Human CD8 T cells in the nasal cavity significantly upregulated PD-1 following RSV infection compared to CD8 T cells from the blood of either healthy or RSV-infected individuals (137). PD-1 and PD-L1 are also both upregulated in the lung tissue following severe infections with either RSV or the 2009 H1N1 IAV pandemic strain (41). *In vitro* human studies have demonstrated that PD-L1 is constitutively expressed on human airway and bronchial epithelial cells, but expression is significantly upregulated following either IAV or RSV infection (136, 139). Similar to *in vivo* mouse studies, *in vitro* PD-L1 blockade resulted in significantly increased CD8 T cell production of IFN-γ, IL-2, and granzyme B following RSV infection (139). Together, these studies demonstrate a critical role for PD-1 in the suppression of CD8 T cell-mediated immunopathology and cytokine production in both mice and humans. In the absence of PD-1 signaling following HMPV infection, CD8 T cell IFN-γ production remains impaired, suggesting the involvement of compensatory inhibitory pathways (140). Antigen-specific lung CD8 T cells express inhibitory receptors Tim-3, LAG-3, and 2B4 following HMPV infection and exhibit enhanced cytokine production following *in vitro* blockade of each receptor individually (140). *In vivo* blockade of LAG-3 partially restored CD8 T cell IFN-γ production in PD-1-deficient mice following HMPV infection (140). Tim-3 has also been demonstrated to be critical in suppressing CD8 T cell responses *in vivo*, as Tim-3 receptor (Galectin-9)-deficient mice exhibited significantly enhanced CD8 T cell responses following both primary and secondary IAV infections (141). Together, these studies indicate that multiple inhibitory receptor pathways are utilized following pulmonary virus infection to dampen the pathogenic CD8 T cell response and prevent immunopathology.

## Concluding Discussion

Successful vaccinations against the majority of respiratory viruses remain elusive. The goal of most vaccination strategies is to induce robust virus-specific neutralizing antibody responses. However, the antibody response generated by infection with many respiratory infections, including RSV and RV, wanes over time. Therefore, neutralizing antibody responses as the sole mediator of a vaccine against most respiratory viruses may not provide long-term protection without yearly vaccination. Vaccination strategies that include the induction of virus-specific CD8 T cell responses, either alone or in combination with humoral immunity, may be advantageous by providing many benefits associated with cellular immune responses. CD8 T cells are critical for the elimination of virus-infected cells, and viral clearance was prolonged in the absence of CD8 T cells following acute respiratory virus infections. Additionally, robust memory CD8 T cell responses efficiently reduced lung viral titers in the absence of neutralizing antibodies following RSV, IAV, or SARS secondary infections. An important property of CD8 T cells is that they often recognize conserved viral proteins, allowing for cross-protection between different virus strains. This is particularly important for heterosubtypic protection of IAV strains, as neutralizing antibodies are not capable of recognizing IAV strains of differing subtypes. Despite their benefits in mediating viral clearance and providing protection against secondary infections, memory CD8 T cell responses have been associated with the induction of immunopathology following respiratory virus infections. The same antiviral mechanisms employed by memory CD8 T cells to accelerate viral clearance also contribute to immunopathology, including the Fas/FasL pathway- and perforin-mediated cytolysis and IFN-γ and TNF cytokine secretion. Thus, the efficient elimination of respiratory viruses by memory CD8 T cells comes at a cost of disease for the host. CD8 T cell-mediated immunopathology appears to be virus-specific. Although high frequency, systemic, antigen-specific memory CD8 T cells induced severe disease and mortality following RSV infection, no pathology was observed using similar systems for IAV and SARS infections. Therefore, induction of memory CD8 T cells as the sole immune mediator may be particularly dangerous for an RSV vaccine, but significantly less so in either an IAV or a SARS vaccine.

**Figure 1:**
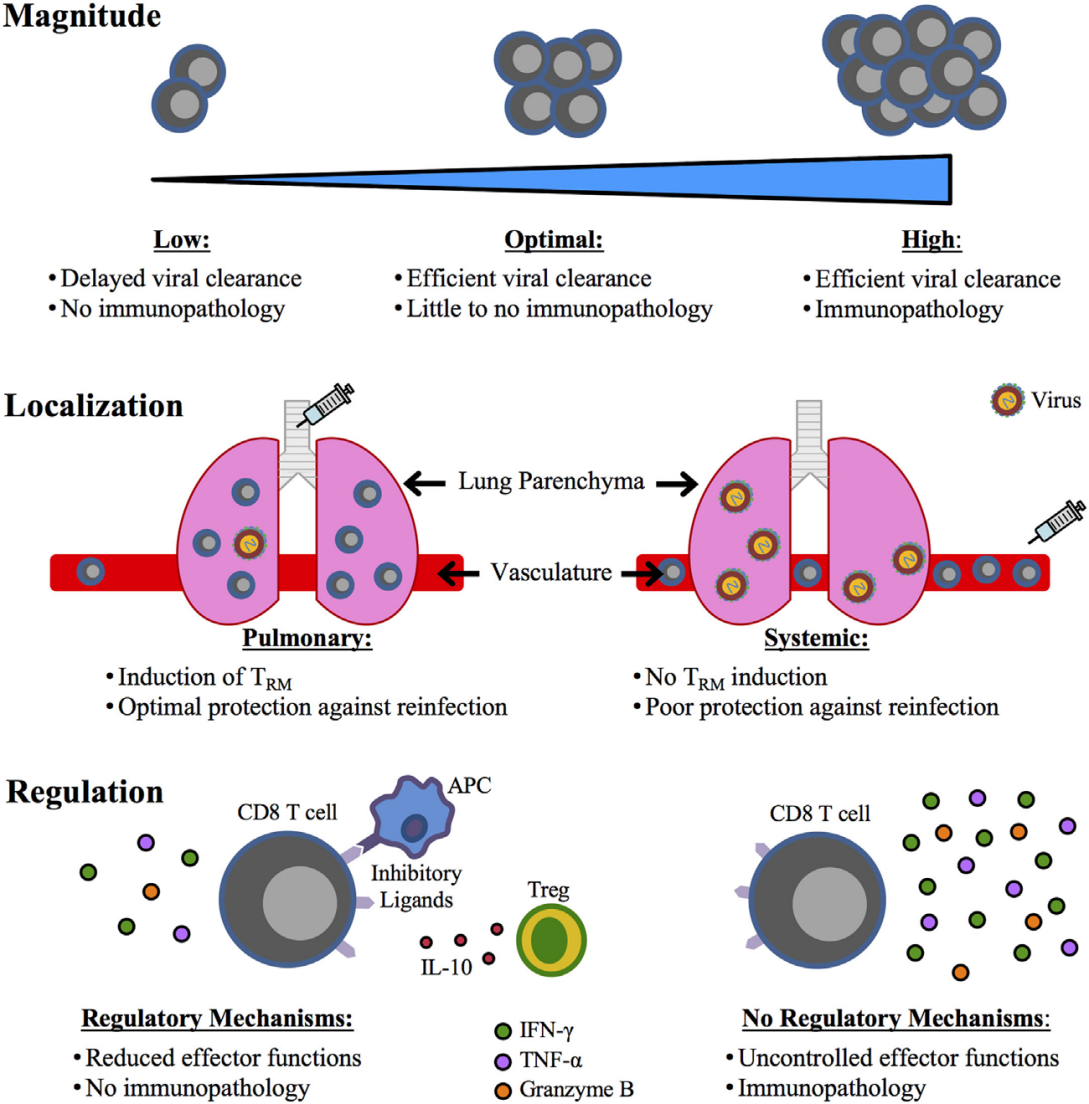
Critical factors for an optimal CD8 T cell-mediated respiratory syncytial virus (RSV) vaccine. A future RSV vaccine designed to elicit a CD8 T cell response will require a balance between CD8 T cell-mediated protection and immunopathology, which may be achieved through the consideration of three important aspects: (1) magnitude, (2) localization, and (3) regulation. An optimal magnitude of the CD8 T cell response will be one that achieves efficient viral clearance in the absence of immunopathology. The vaccination route will be critical in determining the localization of the CD8 T cell response. A pulmonary route of vaccination will induce T_RM_ in the lung that provides superior protection compared to a systemic immunization that would likely not generate protective T_RM_. Lastly, regulation of the CD8 T cell response generated through vaccination will be crucial, as uncontrolled effector functions, particularly IFN-γ production, can result in immunopathology.

To be able to include CD8 T cell responses within a future respiratory virus vaccine, it will be extremely important to determine how to balance CD8 T cell-mediated protection versus immunopathology following respiratory infection. For RSV in particular, three critical aspects to consider in this balance include magnitude, localization, and regulation of the RSV-specific CD8 T cell response (Figure 1). DC-LM immunization generated M2_82-90_-specific CD8 T cells at a frequency of approximately 20% in the peripheral blood, but induced fatal immunopathology following RSV challenge (30). However, DC-prime only and TriVax immunizations generated a much lower frequency of total M2_82_-specific CD8 T cells, and RSV induced significantly reduced disease in these mice (30, 109). Thus, identifying the optimal magnitude of RSV-specific CD8 T cells for protection in the absence of immunopathology is crucial. It is also clear from recent studies in mouse models that localization of RSV-specific CD8 T cells is a significant factor for both their efficacy of mediating viral clearance and their ability to induce immunopathology following infection. Intranasal immunization with MCMV-M generated T_RM_ within the lung tissue that accelerated viral clearance. In contrast, mice administered MCMV-M systemically did not generate T_RM_ and exhibited delayed viral clearance (78). Similar results were observed with local immunization with DC-IAV-M2_82_ (30). M2_82_-specific lung T_RM_ generated by pulmonary immunization did not induce immunopathology following RSV infection, in contrast to systemic DC-LM immunization, which resulted in severe pathology in the absence of T_RM_ cells. Thus, vaccination strategies against RSV will likely be the most effective when administered through a pulmonary route to generate T_RM_ that will provide protection within the lung following reinfection without inducing immunopathology. Lastly, identifying ways to regulate vaccine-generated CD8 T cell responses will likely reduce immunopathology following subsequent infection. IFN-γ produced by CD8 T cells was the primary mediator of immunopathology following RSV infection of DC-LM vaccinated mice (30). However, neutralization of IFN-γ had no effect on lung viral titers, suggesting that CD8 T cells utilize other antiviral mechanisms to mediate viral clearance in this system. Since CD8 T cells are able to reduce viral titers in the absence of IFN-γ, reducing the amount of IFN-γ produced by CD8 T cells would likely result in ameliorated disease following RSV infection. If vaccination strategies can identify mechanisms by which CD8 T cell cytokine production, particularly IFN-γ, can be attenuated without altering their ability to eliminate virus-infected cells, the pathology induced by CD8 T cells would likely also be decreased.

The development of a CD8 T cell-mediated vaccine should be pursued given the limitations of antibody responses to respiratory viruses. It is possible that the ideal vaccine for respiratory virus infections will include the induction of both virus-specific CD8 T cells and neutralizing antibodies. A vaccination approach combining both arms of the adaptive immune response may allow for optimal viral control in the absence of disease symptoms. However, before CD8 T cells can be developed further as a mediator of protective immunity, the balance between protection and pathology must be achieved. Future studies evaluating aspects of memory CD8 T cell magnitude, localization, and regulation will greatly assist in reaching this balance.

## Author Contributions

Both authors wrote and edited the manuscript.

## Conflict of Interest Statement

The authors declare that the research was conducted in the absence of any commercial or financial relationships that could be construed as a potential conflict of interest.

## References

[1] Lessler J Reich NG Brookmeyer R Perl TM Nelson KE Cummings DA. Incubation periods of acute respiratory viral infections: a systematic review. Lancet Infect Dis (2009) 9(5):291–300. 10.1016/S1473-3099(09)70069-6 19393959 PMC4327893

[2] Nair H Nokes DJ Gessner BD Dherani M Madhi SA Singleton RJ Global burden of acute lower respiratory infections due to respiratory syncytial virus in young children: a systematic review and meta-analysis. Lancet (2010) 375(9725):1545–55. 10.1016/S0140-6736(10)60206-1 20399493 PMC2864404

[3] Hall CB Powell KR MacDonald NE Gala CL Menegus ME Suffin SC Respiratory syncytial viral infection in children with compromised immune function. N Engl J Med (1986) 315(2):77–81. 10.1056/NEJM198607103150201 3724802

[4] Falsey AR Cunningham CK Barker WH Kouides RW Yuen JB Menegus M Respiratory syncytial virus and influenza A infections in the hospitalized elderly. J Infect Dis (1995) 172(2):389–94. 10.1093/infdis/172.2.389 7622882

[5] Falsey AR Hennessey PA Formica MA Walsh EE Respiratory syncytial virus infection in elderly and high-risk adults. N Engl J Med (2005) 352(17):1749–59. 10.1056/NEJMoa043951 15858184

[6] Jansen AG Sanders EA Hoes AW van Loon AM Hak E. Influenza- and respiratory syncytial virus-associated mortality and hospitalisations. Eur Respir J (2007) 30(6):1158–66. 10.1183/09031936.00034407 17715167

[7] Reed G Jewett PH Thompson J Tollefson S Wright PF Epidemiology and clinical impact of parainfluenza virus infections in otherwise healthy infants and young children < 5 years old. J Infect Dis (1997) 175(4):807–13. 10.1086/513975 9086134

[8] Henrickson KJ. Parainfluenza viruses. Clin Microbiol Rev (2003) 16(2):242–64. 10.1128/CMR.16.2.242-264.2003 12692097 PMC153148

[9] World Health Organization. Influenza (Seasonal) Fact Sheet. (2017). Available from: http://www.who.int/mediacentre/factsheets/fs211/en/ (Accessed: December, 2017).

[10] Thompson WW Weintraub E Dhankhar P Cheng PY Brammer L Meltzer MI Estimates of US influenza-associated deaths made using four different methods. Influenza Other Respir Viruses (2009) 3(1):37–49. 10.1111/j.1750-2659.2009.00073.x 19453440 PMC4986622

[11] Nair H Brooks WA Katz M Roca A Berkley JA Madhi SA Global burden of respiratory infections due to seasonal influenza in young children: a systematic review and meta-analysis. Lancet (2011) 378(9807):1917–30. 10.1016/S0140-6736(11)61051-9 22078723

[12] Dawood FS Iuliano AD Reed C Meltzer MI Shay DK Cheng PY Estimated global mortality associated with the first 12 months of 2009 pandemic influenza A H1N1 virus circulation: a modelling study. Lancet Infect Dis (2012) 12(9):687–95. 10.1016/S1473-3099(12)70121-4 22738893

[13] Makela MJ Puhakka T Ruuskanen O Leinonen M Saikku P Kimpimaki M Viruses and bacteria in the etiology of the common cold. J Clin Microbiol (1998) 36(2):539–42. 9466772 10.1128/jcm.36.2.539-542.1998 PMC104573

[14] Williams JV Harris PA Tollefson SJ Halburnt-Rush LL Pingsterhaus JM Edwards KM Human metapneumovirus and lower respiratory tract disease in otherwise healthy infants and children. N Engl J Med (2004) 350(5):443–50. 10.1056/NEJMoa025472 14749452 PMC1831873

[15] Kuypers J Martin ET Heugel J Wright N Morrow R Englund JA. Clinical disease in children associated with newly described coronavirus subtypes. Pediatrics (2007) 119(1):e70–6. 10.1542/peds.2006-1406 17130280

[16] Ksiazek TG Erdman D Goldsmith CS Zaki SR Peret T Emery S A novel coronavirus associated with severe acute respiratory syndrome. N Engl J Med (2003) 348(20):1953–66. 10.1056/NEJMoa030781 12690092

[17] Peiris JS Lai ST Poon LL Guan Y Yam LY Lim W Coronavirus as a possible cause of severe acute respiratory syndrome. Lancet (2003) 361(9366):1319–25. 10.1016/S0140-6736(03)13077-2 12711465 PMC7112372

[18] Zaki AM van Boheemen S Bestebroer TM Osterhaus AD Fouchier RA. Isolation of a novel coronavirus from a man with pneumonia in Saudi Arabia. N Engl J Med (2012) 367(19):1814–20. 10.1056/NEJMoa1211721 23075143

[19] Glezen WP Taber LH Frank AL Kasel JA. Risk of primary infection and reinfection with respiratory syncytial virus. Am J Dis Child (1986) 140(6):543–6. 3706232 10.1001/archpedi.1986.02140200053026

[20] Williams JV Wang CK Yang CF Tollefson SJ House FS Heck JM The role of human metapneumovirus in upper respiratory tract infections in children: a 20-year experience. J Infect Dis (2006) 193(3):387–95. 10.1086/499274 16388486 PMC1586246

[21] Zlateva KT de Vries JJ Coenjaerts FE van Loon AM Verheij T Little P Prolonged shedding of rhinovirus and re-infection in adults with respiratory tract illness. Eur Respir J (2014) 44(1):169–77. 10.1183/09031936.00172113 24876172

[22] Butler WT Waldmann TA Rossen RD Douglas RG Jr Couch RB Changes in IgA and IgG concentrations in nasal secretions prior to the appearance of antibody during viral respiratory infection in man. J Immunol (1970) 105(3):584–91. 4318484

[23] Habibi MS Jozwik A Makris S Dunning J Paras A DeVincenzo JP Impaired antibody-mediated protection and defective IgA B-cell memory in experimental infection of adults with respiratory syncytial virus. Am J Respir Crit Care Med (2015) 191(9):1040–9. 10.1164/rccm.201412-2256OC 25730467 PMC4435460

[24] Chen WH Cross AS Edelman R Sztein MB Blackwelder WC Pasetti MF. Antibody and Th1-type cell-mediated immune responses in elderly and young adults immunized with the standard or a high dose influenza vaccine. Vaccine (2011) 29(16):2865–73. 10.1016/j.vaccine.2011.02.017 21352939 PMC3070775

[25] Taylor PM Askonas BA. Influenza nucleoprotein-specific cytotoxic T-cell clones are protective in vivo. Immunology (1986) 58(3):417–20. 2426185 PMC1453480

[26] Graham BS Bunton LA Wright PF Karzon DT. Role of T lymphocyte subsets in the pathogenesis of primary infection and rechallenge with respiratory syncytial virus in mice. J Clin Invest (1991) 88(3):1026–33. 10.1172/JCI115362 1909350 PMC295511

[27] Melendi GA Zavala F Buchholz UJ Boivin G Collins PL Kleeberger SR Mapping and characterization of the primary and anamnestic H-2d-restricted cytotoxic T-lymphocyte response in mice against human metapneumovirus. J Virol (2007) 81(20):11461–7. 10.1128/JVI.02423-06 17670840 PMC2045518

[28] Slutter B Pewe LL Kaech SM Harty JT. Lung airway-surveilling CXCR3(hi) memory CD8+ T cells are critical for protection against influenza A virus. Immunity (2013) 39(5):939–48. 10.1016/j.immuni.2013.09.013 24238342 PMC3872058

[29] Channappanavar R Fett C Zhao J Meyerholz DK Perlman S. Virus-specific memory CD8 T cells provide substantial protection from lethal severe acute respiratory syndrome coronavirus infection. J Virol (2014) 88(19):11034–44. 10.1128/JVI.01505-14 25056892 PMC4178831

[30] Schmidt ME Knudson CJ Hartwig SM Pewe LL Meyerholz DK Langlois RA Memory CD8 T cells mediate severe immunopathology following respiratory syncytial virus infection. PLoS Pathog (2017) 14(1):e1006810 10.1371/journal.ppat.1006810 PMC5766251 29293660

[31] McDermott DS Weiss KA Knudson CJ Varga SM. Central role of dendritic cells in shaping the adaptive immune response during respiratory syncytial virus infection. Future Virol (2011) 6(8):963–73. 10.2217/fvl.11.62 21887154 PMC3163480

[32] Kim TH Lee HK. Differential roles of lung dendritic cell subsets against respiratory virus infection. Immune Netw (2014) 14(3):128–37. 10.4110/in.2014.14.3.128 24999309 PMC4079819

[33] Hufford MM Kim TS Sun J Braciale TJ. The effector T cell response to influenza infection. Curr Top Microbiol Immunol (2015) 386:423–55. 10.1007/82_2014_397 25033753 PMC4224975

[34] Chang J Srikiatkhachorn A Braciale TJ. Visualization and characterization of respiratory syncytial virus F-specific CD8(+) T cells during experimental virus infection. J Immunol (2001) 167(8):4254–60. 10.4049/jimmunol.167.8.4254 11591747

[35] Lukens MV Claassen EA de Graaff PM van Dijk ME Hoogerhout P Toebes M Characterization of the CD8+ T cell responses directed against respiratory syncytial virus during primary and secondary infection in C57BL/6 mice. Virology (2006) 352(1):157–68. 10.1016/j.virol.2006.04.023 16730775

[36] Ruckwardt TJ Bonaparte KL Nason MC Graham BS. Regulatory T cells promote early influx of CD8+ T cells in the lungs of respiratory syncytial virus-infected mice and diminish immunodominance disparities. J Virol (2009) 83(7):3019–28. 10.1128/JVI.00036-09 19153229 PMC2655550

[37] Knudson CJ Weiss KA Hartwig SM Varga SM. The pulmonary localization of virus-specific T lymphocytes is governed by the tissue tropism of infection. J Virol (2014) 88(16):9010–6. 10.1128/JVI.00329-14 24899187 PMC4136240

[38] Flynn KJ Belz GT Altman JD Ahmed R Woodland DL Doherty PC. Virus-specific CD8+ T cells in primary and secondary influenza pneumonia. Immunity (1998) 8(6):683–91. 10.1016/S1074-7613(00)80573-7 9655482

[39] Crowe SR Turner SJ Miller SC Roberts AD Rappolo RA Doherty PC Differential antigen presentation regulates the changing patterns of CD8+ T cell immunodominance in primary and secondary influenza virus infections. J Exp Med (2003) 198(3):399–410. 10.1084/jem.20022151 12885871 PMC2194086

[40] Kolli D Bataki EL Spetch L Guerrero-Plata A Jewell AM Piedra PA T lymphocytes contribute to antiviral immunity and pathogenesis in experimental human metapneumovirus infection. J Virol (2008) 82(17):8560–9. 10.1128/JVI.00699-08 18562525 PMC2519650

[41] Erickson JJ Gilchuk P Hastings AK Tollefson SJ Johnson M Downing MB Viral acute lower respiratory infections impair CD8+ T cells through PD-1. J Clin Invest (2012) 122(8):2967–82. 10.1172/JCI62860 22797302 PMC3408742

[42] van Helden MJ van Kooten PJ Bekker CP Grone A Topham DJ Easton AJ Pre-existing virus-specific CD8+ T-cells provide protection against pneumovirus-induced disease in mice. Vaccine (2012) 30(45):6382–8. 10.1016/j.vaccine.2012.08.027 22940382 PMC3465553

[43] Wiley JA Hogan RJ Woodland DL Harmsen AG Antigen-specific CD8+ T cells persist in the upper respiratory tract following influenza virus infection. J Immunol (2001) 167(6):3293–9. 10.4049/jimmunol.167.6.3293 11544317

[44] Heidema J Rossen JW Lukens MV Ketel MS Scheltens E Kranendonk ME Dynamics of human respiratory virus-specific CD8+ T cell responses in blood and airways during episodes of common cold. J Immunol (2008) 181(8):5551–9. 10.4049/jimmunol.181.8.5551 18832713

[45] de Bree GJ van Leeuwen EM Out TA Jansen HM Jonkers RE van Lier RA Selective accumulation of differentiated CD8+ T cells specific for respiratory viruses in the human lung. J Exp Med (2005) 202(10):1433–42. 10.1084/jem.20051365 16301748 PMC2212987

[46] Heidema J Lukens MV van Maren WW van Dijk ME Otten HG van Vught AJ CD8+ T cell responses in bronchoalveolar lavage fluid and peripheral blood mononuclear cells of infants with severe primary respiratory syncytial virus infections. J Immunol (2007) 179(12):8410–7. 10.4049/jimmunol.179.12.8410 18056387

[47] Hogan RJ Usherwood EJ Zhong W Roberts AA Dutton RW Harmsen AG Activated antigen-specific CD8+ T cells persist in the lungs following recovery from respiratory virus infections. J Immunol (2001) 166(3):1813–22. 10.4049/jimmunol.166.3.1813 11160228

[48] Claassen EA van der Kant PA Rychnavska ZS van Bleek GM Easton AJ van der Most RG. Activation and inactivation of antiviral CD8 T cell responses during murine pneumovirus infection. J Immunol (2005) 175(10):6597–604. 10.4049/jimmunol.175.10.6597 16272314

[49] Jozwik A Habibi MS Paras A Zhu J Guvenel A Dhariwal J RSV-specific airway resident memory CD8+ T cells and differential disease severity after experimental human infection. Nat Commun (2015) 6:10224. 10.1038/ncomms10224 26687547 PMC4703893

[50] Sridhar S Begom S Bermingham A Hoschler K Adamson W Carman W Cellular immune correlates of protection against symptomatic pandemic influenza. Nat Med (2013) 19(10):1305–12. 10.1038/nm.3350 24056771

[51] Steinke JW Liu L Turner RB Braciale TJ Borish L. Immune surveillance by rhinovirus-specific circulating CD4+ and CD8+ T lymphocytes. PLoS One (2015) 10(1):e0115271. 10.1371/journal.pone.0115271 25584821 PMC4293146

[52] Savic M Dembinski JL Kim Y Tunheim G Cox RJ Oftung F Epitope specific T-cell responses against influenza A in a healthy population. Immunology (2016) 147(2):165–77. 10.1111/imm.12548 26489873 PMC4717245

[53] Tzannou I Nicholas SK Lulla P Aguayo-Hiraldo PI Misra A Martinez CA Immunologic profiling of human metapneumovirus for the development of targeted immunotherapy. J Infect Dis (2017) 216(6):678–87. 10.1093/infdis/jix358 28934427 PMC5853664

[54] Kohlmeier JE Reiley WW Perona-Wright G Freeman ML Yager EJ Connor LM Inflammatory chemokine receptors regulate CD8+ T cell contraction and memory generation following infection. J Exp Med (2011) 208(8):1621–34. 10.1084/jem.20102110 21788409 PMC3149221

[55] Chen H Hou J Jiang X Ma S Meng M Wang B Response of memory CD8+ T cells to severe acute respiratory syndrome (SARS) coronavirus in recovered SARS patients and healthy individuals. J Immunol (2005) 175(1):591–8. 10.4049/jimmunol.175.1.591 15972696

[56] Herd KA Nissen MD Hopkins PM Sloots TP Tindle RW. Major histocompatibility complex class I cytotoxic T lymphocyte immunity to human metapneumovirus (hMPV) in individuals with previous hMPV infection and respiratory disease. J Infect Dis (2008) 197(4):584–92. 10.1086/526536 18240952

[57] de Bree GJ Heidema J van Leeuwen EM van Bleek GM Jonkers RE Jansen HM Respiratory syncytial virus-specific CD8+ memory T cell responses in elderly persons. J Infect Dis (2005) 191(10):1710–8. 10.1086/429695 15838799

[58] Ely KH Cauley LS Roberts AD Brennan JW Cookenham T Woodland DL Nonspecific recruitment of memory CD8+ T cells to the lung airways during respiratory virus infections. J Immunol (2003) 170(3):1423–9. 10.4049/jimmunol.170.3.1423 12538703

[59] Kohlmeier JE Miller SC Smith J Lu B Gerard C Cookenham T The chemokine receptor CCR5 plays a key role in the early memory CD8+ T cell response to respiratory virus infections. Immunity (2008) 29(1):101–13. 10.1016/j.immuni.2008.05.011 18617426 PMC2519120

[60] McMaster SR Wilson JJ Wang H Kohlmeier JE Airway-resident memory CD8 T cells provide antigen-specific protection against respiratory virus challenge through rapid IFN-gamma production. J Immunol (2015) 195(1):203–9. 10.4049/jimmunol.1402975 26026054 PMC4475417

[61] Ely KH Roberts AD Woodland DL Cutting edge: effector memory CD8+ T cells in the lung airways retain the potential to mediate recall responses. J Immunol (2003) 171(7):3338–42. 10.4049/jimmunol.171.7.3338 14500625

[62] Kohlmeier JE Cookenham T Roberts AD Miller SC Woodland DL. Type I interferons regulate cytolytic activity of memory CD8+ T cells in the lung airways during respiratory virus challenge. Immunity (2010) 33(1):96–105. 10.1016/j.immuni.2010.06.016 20637658 PMC2908370

[63] Mahnke YD Brodie TM Sallusto F Roederer M Lugli E. The who’s who of T-cell differentiation: human memory T-cell subsets. Eur J Immunol (2013) 43(11):2797–809. 10.1002/eji.201343751 24258910

[64] Gebhardt T Wakim LM Eidsmo L Reading PC Heath WR Carbone FR. Memory T cells in nonlymphoid tissue that provide enhanced local immunity during infection with herpes simplex virus. Nat Immunol (2009) 10(5):524–30. 10.1038/ni.1718 19305395

[65] Turner DL Bickham KL Thome JJ Kim CY D’Ovidio F Wherry EJ Lung niches for the generation and maintenance of tissue-resident memory T cells. Mucosal Immunol (2014) 7(3):501–10. 10.1038/mi.2013.67 24064670 PMC3965651

[66] Wu T Hu Y Lee YT Bouchard KR Benechet A Khanna K Lung-resident memory CD8 T cells (TRM) are indispensable for optimal cross-protection against pulmonary virus infection. J Leukoc Biol (2014) 95(2):215–24. 10.1189/jlb.0313180 24006506 PMC3896663

[67] Takamura S Yagi H Hakata Y Motozono C McMaster SR Masumoto T Specific niches for lung-resident memory CD8+ T cells at the site of tissue regeneration enable CD69-independent maintenance. J Exp Med (2016) 213(13):3057–73. 10.1084/jem.20160938 27815325 PMC5154946

[68] Ostler T Hussell T Surh CD Openshaw P Ehl S. Long-term persistence and reactivation of T cell memory in the lung of mice infected with respiratory syncytial virus. Eur J Immunol (2001) 31(9):2574–82. 10.1002/1521-4141(200109)31:9<2574::AID-IMMU2574>3.0.CO;2-V 11536155

[69] Hogan RJ Cauley LS Ely KH Cookenham T Roberts AD Brennan JW Long-term maintenance of virus-specific effector memory CD8+ T cells in the lung airways depends on proliferation. J Immunol (2002) 169(9):4976–81. 10.4049/jimmunol.169.9.4976 12391211

[70] Ely KH Cookenham T Roberts AD Woodland DL. Memory T cell populations in the lung airways are maintained by continual recruitment. J Immunol (2006) 176(1):537–43. 10.4049/jimmunol.176.1.537 16365448

[71] Kohlmeier JE Miller SC Woodland DL. Cutting edge: antigen is not required for the activation and maintenance of virus-specific memory CD8+ T cells in the lung airways. J Immunol (2007) 178(8):4721–5. 10.4049/jimmunol.178.8.4721 17404250

[72] Khanna KM Aguila CC Redman JM Suarez-Ramirez JE Lefrancois L Cauley LS In situ imaging reveals different responses by naive and memory CD8 T cells to late antigen presentation by lymph node DC after influenza virus infection. Eur J Immunol (2008) 38(12):3304–15. 10.1002/eji.200838602 19009527 PMC2662394

[73] Hu Y Lee YT Kaech SM Garvy B Cauley LS. Smad4 promotes differentiation of effector and circulating memory CD8 T cells but is dispensable for tissue-resident memory CD8 T cells. J Immunol (2015) 194(5):2407–14. 10.4049/jimmunol.1402369 25637015 PMC4337487

[74] Lee YT Suarez-Ramirez JE Wu T Redman JM Bouchard K Hadley GA Environmental and antigen receptor-derived signals support sustained surveillance of the lungs by pathogen-specific cytotoxic T lymphocytes. J Virol (2011) 85(9):4085–94. 10.1128/JVI.02493-10 21345961 PMC3126261

[75] Wakim LM Gupta N Mintern JD Villadangos JA Enhanced survival of lung tissue-resident memory CD8+ T cells during infection with influenza virus due to selective expression of IFITM3. Nat Immunol (2013) 14(3):238–45. 10.1038/ni.2525 23354485

[76] Kinnear E Lambert L McDonald JU Cheeseman HM Caproni LJ Tregoning JS Airway T cells protect against RSV infection in the absence of antibody. Mucosal Immunol (2017) 11(1):249–56. 10.1038/mi.2017.79 28537249

[77] Zens KD Chen JK Farber DL. Vaccine-generated lung tissue-resident memory T cells provide heterosubtypic protection to influenza infection. JCI Insight (2016) 1(10):e85832. 10.1172/jci.insight.85832 27468427 PMC4959801

[78] Morabito KM Ruckwardt TR Redwood AJ Moin SM Price DA Graham BS. Intranasal administration of RSV antigen-expressing MCMV elicits robust tissue-resident effector and effector memory CD8+ T cells in the lung. Mucosal Immunol (2017) 10(2):545–54. 10.1038/mi.2016.48 27220815 PMC5123975

[79] Zens KD Chen JK Guyer RS Wu FL Cvetkovski F Miron M Reduced generation of lung tissue-resident memory T cells during infancy. J Exp Med (2017) 214(10):2915–32. 10.1084/jem.20170521 28855242 PMC5626403

[80] Slutter B Van Braeckel-Budimir N Abboud G Varga SM Salek-Ardakani S Harty JT. Dynamics of influenza-induced lung-resident memory T cells underlie waning heterosubtypic immunity. Sci Immunol (2017) 2(7):eaag2031. 10.1126/sciimmunol.aag2031 28783666 PMC5590757

[81] Lukacher AE Braciale VL Braciale TJ. In vivo effector function of influenza virus-specific cytotoxic T lymphocyte clones is highly specific. J Exp Med (1984) 160(3):814–26. 10.1084/jem.160.3.814 6206190 PMC2187390

[82] Cannon MJ Openshaw PJ Askonas BA. Cytotoxic T cells clear virus but augment lung pathology in mice infected with respiratory syncytial virus. J Exp Med (1988) 168(3):1163–8. 10.1084/jem.168.3.1163 3262705 PMC2189034

[83] Alwan WH Record FM Openshaw PJ. CD4+ T cells clear virus but augment disease in mice infected with respiratory syncytial virus. Comparison with the effects of CD8+ T cells. Clin Exp Immunol (1992) 88(3):527–36. 10.1111/j.1365-2249.1992.tb06482.x 1351433 PMC1554527

[84] Alwan WH Kozlowska WJ Openshaw PJ. Distinct types of lung disease caused by functional subsets of antiviral T cells. J Exp Med (1994) 179(1):81–9. 10.1084/jem.179.1.81 8270885 PMC2191312

[85] Yap KL Ada GL. The recovery of mice from influenza virus infection: adoptive transfer of immunity with immune T lymphocytes. Scand J Immunol (1978) 7(5):389–97. 10.1111/j.1365-3083.1978.tb00469.x 307273

[86] Yap KL Ada GL McKenzie IF Transfer of specific cytotoxic T lymphocytes protects mice inoculated with influenza virus. Nature (1978) 273(5659):238–9. 10.1038/273238a0 306072

[87] Wells MA Ennis FA Albrecht P Recovery from a viral respiratory infection. II. Passive transfer of immune spleen cells to mice with influenza pneumonia. J Immunol (1981) 126(3):1042–6. 7462625

[88] Cannon MJ Stott EJ Taylor G Askonas BA. Clearance of persistent respiratory syncytial virus infections in immunodeficient mice following transfer of primed T cells. Immunology (1987) 62(1):133–8. 3498683 PMC1453729

[89] Ostler T Davidson W Ehl S Virus clearance and immunopathology by CD8(+) T cells during infection with respiratory syncytial virus are mediated by IFN-gamma. Eur J Immunol (2002) 32(8):2117–23. 10.1002/1521-4141(200208)32:8<2117::AID-IMMU2117>3.0.CO;2-C 12209623

[90] Taylor G Stott EJ Hayle AJ. Cytotoxic lymphocytes in the lungs of mice infected with respiratory syncytial virus. J Gen Virol (1985) 66(Pt 12):2533–8. 10.1099/0022-1317-66-12-2533 2866227

[91] Fishaut M Tubergen D McIntosh K Cellular response to respiratory viruses with particular reference to children with disorders of cell-mediated immunity. J Pediatr (1980) 96(2):179–86. 10.1016/S0022-3476(80)80799-2 6243354

[92] El Saleeby CM Suzich J Conley ME DeVincenzo JP. Quantitative effects of palivizumab and donor-derived T cells on chronic respiratory syncytial virus infection, lung disease, and fusion glycoprotein amino acid sequences in a patient before and after bone marrow transplantation. Clin Infect Dis (2004) 39(2):e17–20. 10.1086/421779 15307047

[93] McMichael AJ Gotch FM Noble GR Beare PA. Cytotoxic T-cell immunity to influenza. N Engl J Med (1983) 309(1):13–7. 10.1056/NEJM198307073090103 6602294

[94] Wang Z Wan Y Qiu C Quinones-Parra S Zhu Z Loh L Recovery from severe H7N9 disease is associated with diverse response mechanisms dominated by CD8(+) T cells. Nat Commun (2015) 6:6833 10.1038/ncomms7833 25967273 PMC4479016

[95] Harty JT Tvinnereim AR White DW. CD8+ T cell effector mechanisms in resistance to infection. Annu Rev Immunol (2000) 18:275–308. 10.1146/annurev.immunol.18.1.275 10837060

[96] Topham DJ Tripp RA Doherty PC. CD8+ T cells clear influenza virus by perforin or Fas-dependent processes. J Immunol (1997) 159(11):5197–200. 9548456

[97] Hamada H Bassity E Flies A Strutt TM Garcia-Hernandez Mde L McKinstry KK Multiple redundant effector mechanisms of CD8+ T cells protect against influenza infection. J Immunol (2013) 190(1):296–306. 10.4049/jimmunol.1200571 23197262 PMC3864858

[98] Ishikawa E Nakazawa M Yoshinari M Minami M. Role of tumor necrosis factor-related apoptosis-inducing ligand in immune response to influenza virus infection in mice. J Virol (2005) 79(12):7658–63. 10.1128/JVI.79.12.7658-7663.2005 15919918 PMC1143624

[99] Brincks EL Katewa A Kucaba TA Griffith TS Legge KL. CD8 T cells utilize TRAIL to control influenza virus infection. J Immunol (2008) 181(7):4918–25. 10.4049/jimmunol.181.10.7428-a 18802095 PMC2610351

[100] Aung S Rutigliano JA Graham BS. Alternative mechanisms of respiratory syncytial virus clearance in perforin knockout mice lead to enhanced disease. J Virol (2001) 75(20):9918–24. 10.1128/JVI.75.20.9918-9924.2001 11559824 PMC114563

[101] Rutigliano JA Graham BS. Prolonged production of TNF-alpha exacerbates illness during respiratory syncytial virus infection. J Immunol (2004) 173(5):3408–17. 10.4049/jimmunol.173.5.3408 15322205

[102] Hussell T Pennycook A Openshaw PJ. Inhibition of tumor necrosis factor reduces the severity of virus-specific lung immunopathology. Eur J Immunol (2001) 31(9):2566–73. 10.1002/1521-4141(200109)31:9<2566::AID-IMMU2566>3.0.CO;2-L 11536154

[103] Frey S Krempl CD Schmitt-Graff A Ehl S. Role of T cells in virus control and disease after infection with pneumonia virus of mice. J Virol (2008) 82(23):11619–27. 10.1128/JVI.00375-08 18815308 PMC2583671

[104] Wiley JA Cerwenka A Harkema JR Dutton RW Harmsen AG Production of interferon-gamma by influenza hemagglutinin-specific CD8 effector T cells influences the development of pulmonary immunopathology. Am J Pathol (2001) 158(1):119–30. 10.1016/S0002-9440(10)63950-8 11141485 PMC1850251

[105] Durbin JE Johnson TR Durbin RK Mertz SE Morotti RA Peebles RS The role of IFN in respiratory syncytial virus pathogenesis. J Immunol (2002) 168(6):2944–52. 10.4049/jimmunol.168.6.2944 11884466

[106] Lee JY Chang J Recombinant baculovirus-based vaccine expressing M2 protein induces protective CD8+ T-cell immunity against respiratory syncytial virus infection. J Microbiol (2017) 55(11):900–8. 10.1007/s12275-017-7306-6 29076066

[107] Wen SC Schuster JE Gilchuk P Boyd KL Joyce S Williams JV. Lung CD8+ T cell impairment occurs during human metapneumovirus infection despite virus-like particle induction of functional CD8+ T cells. J Virol (2015) 89(17):8713–26. 10.1128/JVI.00670-15 26063431 PMC4524081

[108] Herd KA Mahalingam S Mackay IM Nissen M Sloots TP Tindle RW. Cytotoxic T-lymphocyte epitope vaccination protects against human metapneumovirus infection and disease in mice. J Virol (2006) 80(4):2034–44. 10.1128/JVI.80.4.2034-2044.2006 16439559 PMC1367143

[109] Lee S Stokes KL Currier MG Sakamoto K Lukacs NW Celis E Vaccine-elicited CD8+ T cells protect against respiratory syncytial virus strain A2-line19F-induced pathogenesis in BALB/c mice. J Virol (2012) 86(23):13016–24. 10.1128/JVI.01770-12 23015695 PMC3497630

[110] Schulman JL Kilbourne ED. Induction of partial specific heterotypic immunity in mice by a single infection with influenza a virus. J Bacteriol (1965) 89:170–4. 14255658 10.1128/jb.89.1.170-174.1965 PMC315565

[111] Liang S Mozdzanowska K Palladino G Gerhard W. Heterosubtypic immunity to influenza type A virus in mice. Effector mechanisms and their longevity. J Immunol (1994) 152(4):1653–61. 8120375

[112] Christensen JP Doherty PC Branum KC Riberdy JM. Profound protection against respiratory challenge with a lethal H7N7 influenza A virus by increasing the magnitude of CD8+ T-cell memory. J Virol (2000) 74(24):11690–6. 10.1128/JVI.74.24.11690-11696.2000 11090168 PMC112451

[113] Seo SH Peiris M Webster RG. Protective cross-reactive cellular immunity to lethal A/Goose/Guangdong/1/96-like H5N1 influenza virus is correlated with the proportion of pulmonary CD8+ T cells expressing gamma interferon. J Virol (2002) 76(10):4886–90. 10.1128/JVI.76.10.4886-4890.2002 11967305 PMC136145

[114] Weinfurter JT Brunner K Capuano SV III Li C Broman KW Kawaoka Y Cross-reactive T cells are involved in rapid clearance of 2009 pandemic H1N1 influenza virus in nonhuman primates. PLoS Pathog (2011) 7(11):e1002381. 10.1371/journal.ppat.1002381 22102819 PMC3213121

[115] Hayward AC Wang L Goonetilleke N Fragaszy EB Bermingham A Copas A Natural T cell-mediated protection against seasonal and pandemic influenza. Results of the flu watch cohort study. Am J Respir Crit Care Med (2015) 191(12):1422–31. 10.1164/rccm.201411-1988OC 25844934 PMC4476562

[116] Bont L Versteegh J Swelsen WT Heijnen CJ Kavelaars A Brus F Natural reinfection with respiratory syncytial virus does not boost virus-specific T-cell immunity. Pediatr Res (2002) 52(3):363–7. 10.1203/00006450-200209000-00009 12193668

[117] Bender BS Croghan T Zhang L Small PA Jr. Transgenic mice lacking class I major histocompatibility complex-restricted T cells have delayed viral clearance and increased mortality after influenza virus challenge. J Exp Med (1992) 175(4):1143–5. 10.1084/jem.175.4.1143 1552285 PMC2119177

[118] Tregoning JS Pribul PK Pennycook AM Hussell T Wang B Lukacs N The chemokine MIP1alpha/CCL3 determines pathology in primary RSV infection by regulating the balance of T cell populations in the murine lung. PLoS One (2010) 5(2):e9381. 10.1371/journal.pone.0009381 20195359 PMC2827540

[119] Walsh KB Teijaro JR Brock LG Fremgen DM Collins PL Rosen H Animal model of respiratory syncytial virus: CD8+ T cells cause a cytokine storm that is chemically tractable by sphingosine-1-phosphate 1 receptor agonist therapy. J Virol (2014) 88(11):6281–93. 10.1128/JVI.00464-14 24672024 PMC4093868

[120] Bem RA Bos AP Bots M Wolbink AM van Ham SM Medema JP Activation of the granzyme pathway in children with severe respiratory syncytial virus infection. Pediatr Res (2008) 63(6):650–5. 10.1203/PDR.0b013e31816fdc32 18317234 PMC7100119

[121] Welliver TP Garofalo RP Hosakote Y Hintz KH Avendano L Sanchez K Severe human lower respiratory tract illness caused by respiratory syncytial virus and influenza virus is characterized by the absence of pulmonary cytotoxic lymphocyte responses. J Infect Dis (2007) 195(8):1126–36. 10.1086/512615 17357048 PMC7109876

[122] Welliver TP Reed JL Welliver RC Sr. Respiratory syncytial virus and influenza virus infections: observations from tissues of fatal infant cases. Pediatr Infect Dis J (2008) 27(10 Suppl):S92–6. 10.1097/INF.0b013e318168b706 18820587

[123] Mejias A Dimo B Suarez NM Garcia C Suarez-Arrabal MC Jartti T Whole blood gene expression profiles to assess pathogenesis and disease severity in infants with respiratory syncytial virus infection. PLoS Med (2013) 10(11):e1001549. 10.1371/journal.pmed.1001549 24265599 PMC3825655

[124] Chang J Braciale TJ. Respiratory syncytial virus infection suppresses lung CD8+ T-cell effector activity and peripheral CD8+ T-cell memory in the respiratory tract. Nat Med (2002) 8(1):54–60. 10.1038/nm0102-54 11786907

[125] Vallbracht S Unsold H Ehl S. Functional impairment of cytotoxic T cells in the lung airways following respiratory virus infections. Eur J Immunol (2006) 36(6):1434–42. 10.1002/eji.200535642 16708402

[126] DiNapoli JM Murphy BR Collins PL Bukreyev A. Impairment of the CD8+ T cell response in lungs following infection with human respiratory syncytial virus is specific to the anatomical site rather than the virus, antigen, or route of infection. Virol J (2008) 5:105. 10.1186/1743-422X-5-105 18816384 PMC2561024

[127] Fulton RB Olson MR Varga SM. Regulation of cytokine production by virus-specific CD8 T cells in the lungs. J Virol (2008) 82(16):7799–811. 10.1128/JVI.00840-08 18524828 PMC2519597

[128] Fulton RB Meyerholz DK Varga SM. Foxp3+ CD4 regulatory T cells limit pulmonary immunopathology by modulating the CD8 T cell response during respiratory syncytial virus infection. J Immunol (2010) 185(4):2382–92. 10.4049/jimmunol.1000423 20639494 PMC2923480

[129] Betts RJ Prabhu N Ho AW Lew FC Hutchinson PE Rotzschke O Influenza A virus infection results in a robust, antigen-responsive, and widely disseminated Foxp3+ regulatory T cell response. J Virol (2012) 86(5):2817–25. 10.1128/JVI.05685-11 22205730 PMC3302292

[130] Brincks EL Roberts AD Cookenham T Sell S Kohlmeier JE Blackman MA Antigen-specific memory regulatory CD4+Foxp3+ T cells control memory responses to influenza virus infection. J Immunol (2013) 190(7):3438–46. 10.4049/jimmunol.1203140 23467933 PMC3608733

[131] Liu J Ruckwardt TJ Chen M Nicewonger JD Johnson TR Graham BS. Epitope-specific regulatory CD4 T cells reduce virus-induced illness while preserving CD8 T-cell effector function at the site of infection. J Virol (2010) 84(20):10501–9. 10.1128/JVI.00963-10 20686045 PMC2950556

[132] Sun J Madan R Karp CL Braciale TJ. Effector T cells control lung inflammation during acute influenza virus infection by producing IL-10. Nat Med (2009) 15(3):277–84. 10.1038/nm.1929 19234462 PMC2693210

[133] Weiss KA Christiaansen AF Fulton RB Meyerholz DK Varga SM. Multiple CD4+ T cell subsets produce immunomodulatory IL-10 during respiratory syncytial virus infection. J Immunol (2011) 187(6):3145–54. 10.4049/jimmunol.1100764 21844390 PMC3304096

[134] Loebbermann J Schnoeller C Thornton H Durant L Sweeney NP Schuijs M IL-10 regulates viral lung immunopathology during acute respiratory syncytial virus infection in mice. PLoS One (2012) 7(2):e32371. 10.1371/journal.pone.0032371 22393401 PMC3290561

[135] Zou Q Wu B Xue J Fan X Feng C Geng S CD8+ Treg cells suppress CD8+ T cell-responses by IL-10-dependent mechanism during H5N1 influenza virus infection. Eur J Immunol (2014) 44(1):103–14. 10.1002/eji.201343583 24114149 PMC4165276

[136] McNally B Ye F Willette M Flano E. Local blockade of epithelial PDL-1 in the airways enhances T cell function and viral clearance during influenza virus infection. J Virol (2013) 87(23):12916–24. 10.1128/JVI.02423-13 24067957 PMC3838157

[137] Yao S Jiang L Moser EK Jewett LB Wright J Du J Control of pathogenic effector T-cell activities in situ by PD-L1 expression on respiratory inflammatory dendritic cells during respiratory syncytial virus infection. Mucosal Immunol (2015) 8(4):746–59. 10.1038/mi.2014.106 25465101 PMC4632244

[138] Erickson JJ Rogers MC Hastings AK Tollefson SJ Williams JV Programmed death-1 impairs secondary effector lung CD8+ T cells during respiratory virus reinfection. J Immunol (2014) 193(10):5108–17. 10.4049/jimmunol.1302208 25339663 PMC4225166

[139] Telcian AG Laza-Stanca V Edwards MR Harker JA Wang H Bartlett NW RSV-induced bronchial epithelial cell PD-L1 expression inhibits CD8+ T cell nonspecific antiviral activity. J Infect Dis (2011) 203(1):85–94. 10.1093/infdis/jiq020 21148500 PMC3086441

[140] Erickson JJ Rogers MC Tollefson SJ Boyd KL Williams JV. Multiple inhibitory pathways contribute to lung CD8+ T cell impairment and protect against immunopathology during acute viral respiratory infection. J Immunol (2016) 197(1):233–43. 10.4049/jimmunol.1502115 27259857 PMC4933524

[141] Sharma S Sundararajan A Suryawanshi A Kumar N Veiga-Parga T Kuchroo VK T cell immunoglobulin and mucin protein-3 (tim-3)/galectin-9 interaction regulates influenza A virus-specific humoral and CD8 T-cell responses. Proc Natl Acad Sci U S A (2011) 108(47):19001–6. 10.1073/pnas.1107087108 22052881 PMC3223445

